# A Case Report of Recurrent Primary Pituitary Abscess: Challenges in Diagnosis and Treatment

**DOI:** 10.2174/0118715303338996241021094336

**Published:** 2025-01-08

**Authors:** Dilan Ozaydin, Ahmet Numan Demir, Tufan Agah Kartum, Ender Vergili, Pinar Kadioglu, Necmettin Tanriover

**Affiliations:** 1 Department of Neurosurgery, Kartal Dr Lutfi Kirdar City Hospital, Health Sciences University, Istanbul, Turkey;; 2 Department of Endocrinology, Metabolism, and Diabetes, Istanbul University-Cerrahpasa, Istanbul, Turkey;; 3 Department of Neurosurgery, Istanbul University-Cerrahpaşa, Istanbul, Turkey

**Keywords:** Diabetes insipidus, hypopituitarism, primary pituitary abscess, recurrent, visual field deficit, endoscopic endonasal transsphenoidal surgery

## Abstract

**Background:**

Primary pituitary abscess is a rare disease with no specific symptoms for pituitary abscess alone. A preoperative diagnosis is quite challenging due to unclear imaging findings.

**Case Presentation:**

We report the case of a patient with a pituitary lesion who presented with hypopituitarism, diabetes insipidus, and visual field defect and was misdiagnosed as a possible cystic pituitary adenoma. Endoscopic endonasal transsphenoidal surgery (ETSS) was performed, and surprisingly, only pus was found, and complete resection of the lesion was achieved. Coagulase-negative staphylococci were detected in the culture, and appropriate antibiotic therapy was administered for six weeks. Diabetes insipidus and hypopituitarism did not improve. One year later, the abscess recurred, and a second operation with complete resection was performed.

**Conclusion:**

Knowledge of primary pituitary abscess, a rare infectious disease, is essential for early detection and successful treatment. Most patients have a chronic and silent prediagnostic course with symptoms that are not specific to pituitary abscess alone. The primary treatment option is EETS, followed by long-term, relevant antibiotics. The disease can be resistant and recur despite appropriate treatment, especially in patients with risk factors. Therefore, long-term follow-up of patients is essential.

## 1 INTRODUCTION

A pituitary abscess is a rare disease in which purulent material accumulates in the pituitary gland. It accounts for less than 1% of all pituitary lesions [1]. Only a few hundred patients can be found in the literature, most of them in the form of case reports [1]. If the disease occurs in intact pituitary tissue, it is referred to as a primary pituitary abscess [2]. It is assumed to develop due to pre-existing lesions in the pituitary region, such as pituitary adenoma, Rathke's cleft cysts, craniopharyngioma, or infections. In this case, one speaks of a secondary pituitary abscess. A pituitary abscess is usually characterized by an enlarged pituitary mass and associated endocrine dysfunction. It is difficult to diagnose before surgery because the clinical symptoms are similar to those of other pituitary lesions [3].

In most cases, the diagnosis of a pituitary abscess is made intraoperatively/postoperatively or incidentally at autopsy. We present a case of an intraoperatively diagnosed primary pituitary abscess that recurred after one year of follow-up.

## 2 CASE PRESENTATION

**Fig. (1):**
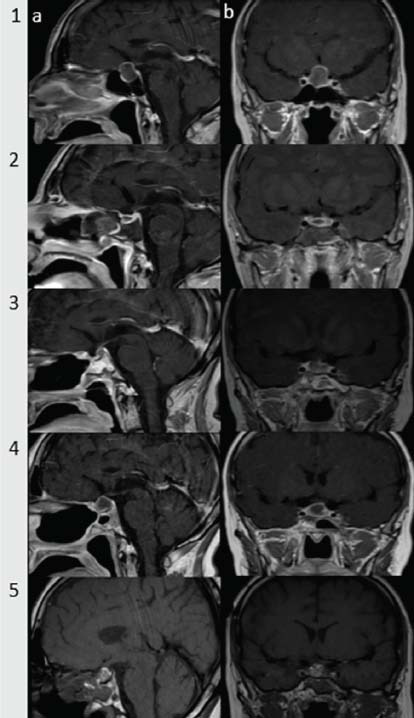
Preoperative sella MRI, sagittal and coronal plane. (**a**) T1-weighted hyperintense lesion with peripheral rim-like contrast enhancement was observed in the sella cavity. It measured approximately 2 cm and filled the cavity, slightly elevating the chiasm and extending towards the suprasellar cistern. The lesion showed minimal protrusion of the sphenoid sinus and encircled the internal carotid artery at an angle of approximately 180º, extending into the left cavernous sinus. (**b**) MRI of early postoperative sella, sagittal and coronal plane. (**c**) MRI of the Sella in the third month after surgery, sagittal and coronal plane. (**d**) MRI of the Sella in the first postoperative year, sagittal and coronal plane. (**e**) MRI of the second ETSS, early postoperative, sagittal, and coronal plane.

Written informed consent was obtained from the patient to participate in the study and to publish the data. A 67-year-old woman complained of severe headache, nausea, vomiting and weakness. She also complained that she drank 7-8 liters of water daily and was polyuric. Secondary adrenal insufficiency was diagnosed when cortisol and adrenocorticotropic hormone levels were deficient. Magnetic resonance imaging (MRI) of the sella region was performed; a T1-weighted (W) hyperintense lesion with peripheral rim-like contrast enhancement was noted in the sella cavity, measuring approximately 2 cm, filling the cavity, slightly elevating the chiasm and extending towards the suprasellar cistern (Fig. **1a**). There was no known medical history of disease or drug use, and she was a non-smoker and did not drink alcohol.

On physical examination, the patient showed no fever. Her blood pressure was 120/80 mmHg, and her pulse was regular at 70 beats per minute. There were no meningeal signs and no neurological or cranial nerve deficits. The patient was postmenopausal and had no galactorrhea. During the initial testing of the patient, urine specific gravity was low at 1.004, and acute phase reactants were not elevated. The evaluation of the pituitary hormones of the patient is shown in Table **1**. The examination revealed secondary adrenal insufficiency and secondary hypothyroidism. Diabetes insipidus (DI) was investigated due to complaints of polyuria and polydipsia. The patient, who received an appropriate thyroid and corticosteroid replacement dose, underwent a fluid restriction test. During the test, the patient was weighed every hour. In addition, urine volume and osmolality, as well as plasma osmolality, were measured every hour. About 4 hours after the start of the test, the patient's serum sodium was 147 meq/L, plasma osmolality was 305 mOsm/kg, urine osmolality was 250 mOsm/kg, and she had lost 3% (1.6 kg) of her initial weight. The patient received 2 mcg of desmopressin IV. After administration of desmopressin, the urine osmolality increased to 750 mOsm/kg after 2 hours. The patient was then diagnosed with central DI, and desmopressin substitution was initiated. Humphrey's visual field examination revealed bitemporal hemianopsia. Cranial computed tomography of the patient showed no calcification of the pituitary mass, and a craniopharyngioma was ruled out.

The patient was preoperatively diagnosed with a possible non-functional pituitary adenoma or cystic prolactinoma. Due to the existing visual field defect, endoscopic endonasal transsphenoidal surgery (EETS) was planned after appropriate replacement therapies. During the EETS, a yellow-green purulent fluid leaked from the sella when the dura was opened. The contents of the possible abscess were removed for pathologic and microbiologic examination, aspirated, and the wall resected. Postoperative MRI examination of the sella after 24 hours revealed that the central part of the sella cavity was defective, the entire abscess was removed, and no debris was found (Fig. **1b**). Microscopic examination of the removed material revealed many leukocytes and coagulase-negative staphylococci were detected in the culture. Mycological examination of the aspirate was negative. No pathogen was isolated in the blood culture. Pathologic examination revealed normal adenohypophyseal tissue, exudate rich in polymorphonuclear leukocytes, acellular proteinaceous material, and clots. Hormone replacement therapy was continued in the patient, who had no postoperative fever. Intravenous treatment with ceftriaxone and metronidazole was continued for four weeks. The patient was discharged after being treated orally with cefixime and metronidazole for two weeks.

At the follow-up examination after three months, the patient was asymptomatic. Panhypopituitarism and diabetes insipidus persisted. MRI of the sella showed a 7 mm peripheral thin rim enhancement lesion, which was considered suspicious for a recurrent abscess. The pituitary stalk was slightly displaced to the left (Fig. **1c**). Visual field examination was regular. No findings were noted on neurological examination. Follow-up examinations were recommended. When the patient came for a follow-up examination in the first year, she was still asymptomatic and continued to suffer from panhypopituitarism and diabetes insipidus. MRI of the sella revealed progression of the suspicious appearance in the form of a recurrent abscess (Fig. **1d**). There were no findings on neurological examination. There was no increase in the acute phase. Blood cultures were taken from the patient without fever, and no growth was detected. The patient underwent a second operation as the sellar lesion had enlarged, most likely indicating the recurrence of a primary pituitary abscess. A complete resection was performed *via* the EETS, and postoperative MRI of the sella showed no residual lesion in the initial period (Fig. **1e**). Navigation screenshots were taken during the procedure and are shown in Fig. (**2**). Microscopic examination of the sample taken from the abscess revealed a small number of leukocytes, but no pathogen was isolated in the culture. The patient was treated with oral cefixime for two weeks. Hormone replacement therapy was continued. The patient, whose general condition was good, was discharged for follow-up care.

## 3 DISCUSSION

We present a rare case of primary pituitary abscess that presented with headache, panhypopituitarism, and diabetes insipidus and was treated with EETS and antibiotics but then required reoperation due to recurrence.

Almost 70% of all pituitary abscesses can develop primarily in a previously normal pituitary gland, as in our case [1]. Pathologies of the pituitary gland, such as craniopharyngioma, pituitary adenoma, and Rathke's cleft cyst, as well as immunosuppression, previous cranial irradiation, or surgical procedures on the pituitary gland, can cause the development of a secondary abscess [2, 3]. Although there are several hypotheses about how pathogens cause abscesses in healthy pituitary glands, the pathophysiology of primary pituitary abscesses is still unknown [4-7]. Possible mechanisms include hematogenous or direct spread from a neighboring site of infection, such as sphenoid sinusitis or tooth extraction. However, only 3-17% of patients with a primary pituitary abscess are reported in the literature to have sepsis or a neighboring infection [4-7]. We tried to find such history in the case presented here. However, the insidious and sluggish nature of the disease, the long interval between the disease and presentation, and the patient's presentation to different centers may have prevented such a possible focus from being discovered.

Preoperative diagnosis may be difficult as a primary pituitary abscess is rare, and the symptoms occur in all sellar lesions and are not specific to abscesses. Many neurosurgeons have never dealt with pituitary abscesses in their professional lives. Therefore, it can be challenging to consider this entity in the differential diagnosis of sellar mass lesions. The most common symptom of pituitary abscesses is headache, with a rate of 70-100% [8]. As with our patients, many patients have no fever, leukocytosis, or meningismus, which is one of the reasons why abscesses are not considered [4, 6, 9-11]. Visual disturbances have been reported in 20-100% of cases, primarily due to mass effect [12, 13]. Dysfunction of the anterior pituitary is observed in about 70-85% of patients, while diabetes insipidus has been reported in 25% [2]. Multiple and non-specific symptoms complicate the diagnosis of primary pituitary abscess, resulting in patients being examined by many different specialties, leading to misdiagnosis or a significant delay in diagnosis.

Preoperative imaging is an essential step in the characterization of pituitary lesions. The preferred imaging modality is sella MRI. The typical appearance of a pituitary abscess is an iso- or hypointense cystic lesion on T1W and T2W MRI. Peripheral ring-shaped contrast enhancement may be seen after contrast administration. Similar features may also be seen in other sellar lesions, such as craniopharyngiomas and Rathke's cleft cysts. Computed tomography can be used for differential diagnosis in calcification, as it supports a craniopharyngioma. However, there is no specific diagnostic imaging finding.

Transsphenoidal surgery of the lesion with decompression of the Sella is the most effective and safest method in patients with a mass effect [1-13]. This should be followed by appropriate antibiotic therapy for 4-6 weeks. Gram-positive cocci have been identified as the causative agent in half of the cases described in the literature [2, 4-6, 9, 10, 14, 15]. In our case, Gram-positive and coagulase-negative cocci were defined as pathogens. According to the sensitivity of the antibiogram, the pathogen was treated with antibiotics for six weeks.

The visual findings improve in most cases with pituitary abscesses when the compression on the optic nerve apparatus is surgically removed, as in our case [1-13]. Diabetes insipidus is a rare symptom of pituitary adenomas. Therefore, preoperative diabetes insipidus is a valuable finding in a primary pituitary abscess [2]. In our case, all other anterior pituitary hormones and antidiuretic hormones were deficient except prolactin. This indicates partial damage to the pituitary gland. At the very least, it shows healthy mammotrophic cells in the pituitary gland. Hyperprolactinemia was observed in our case due to the blockade of dopaminergic inhibition caused by the pituitary abscess pressing on the pituitary stalk. Pituitary abscesses have been reported in the literature to cause pituitary hormone deficiency to varying degrees. In general, panhypopituitarism is reported in 40%, diabetes insipidus in 25%, deficiency of one or two anterior pituitary hormones in 35%, and normal endocrine function of the pituitary in 15% [2]. This situation depends on the abscess size, the amount of pituitary tissue involved, and the duration of exposure. Hyperprolactinemia due to pituitary stalk compression, as in our case, has been demonstrated in a few cases. In a systematic review of 488 cases, hyperprolactinemia was reported in 10 cases [1]. In these cases, stalk compression was also discussed as a possible cause. No association has been reported between hyperprolactinemia and any clinical features, radiological findings, other hormone deficiencies, or recurrence. Future analyses of increasing patient numbers in the literature will be helpful in this issue. The improvement in pituitary hormones after treatment of a pituitary abscess is reported in the literature to be around 30% [2]. The factors that affect hormone recovery are related to both the surgery and the factors mentioned above that cause hormone deficiency. Maximal resection of the lesion while preserving healthy pituitary tissue can provide hormonal recovery. In our case, it was observed that healthy pituitary tissue was preserved after surgery, and preoperative prolactin secretion continued in the postoperative period. In addition, the increase in ACTH levels during the follow-up period in our case indicates a possible recovery of corticotroph cells. Hormonal evaluation will continue during the follow-up.

Antibiotic therapy without surgical intervention for pituitary abscesses remains controversial [2]. The benefit of antibiotic administration for purulent material that has yet to be systematically seeded and is surrounded by a wall is limited. Furthermore, the infectious material must be examined to determine the type of pathogen and the effective antibiotic. In the case we presented, no antibiotic therapy was administered as there were no clinical and laboratory signs of infection three months after the first surgery, and no pathogen could be detected in the blood culture. Since the patient was asymptomatic and the radiologic examination indicated a suspicious sub-centimetric recurrence, it was decided to observe the patient for a while longer. A second operation was decided when the suspicious recurrent abscess increased in size on subsequent follow-up. However, it is still unclear whether antibiotic therapy or early re-operation was necessary in our case three months after the first surgery, confirming the need for evidence-based treatment protocols for these patients.

In our case, a recurrence was detected after a one-year follow-up period, and she underwent surgery again. The recurrence rate of a pituitary abscess is given in the literature as 10-20% [6, 11]. The recurrence times are between three and 36 months [6, 11]. The surgical technique, insufficient duration and extent of antibiotic therapy, and patient immunosuppression are cited as risk factors for recurrence [1-13]. The MRI of the sella, which was performed on the 24^th^ postoperative hour, did not reveal any residual abscess in our patient. Intravenous antibiotics were administered for four weeks, and oral antibiotics for two weeks postoperatively. Our patient was 67 years old and had no diseases, such as diabetes or chronic viral infections, that could cause immunosuppression. However, hypopituitarism did not improve in our patient postoperatively. We could not identify any risk factors for recurrence other than possibly advanced age and persistent hypopituitarism.

## CONCLUSION

Knowledge of primary pituitary abscess, a rare infectious disease, is essential for early detection and successful treatment, as most patients have a chronic and silent prediagnostic course with symptoms that are not specific to pituitary abscess alone. The primary treatment option is EETS, followed by long-term, relevant antibiotics. While compression-related symptoms and signs usually improve after surgery, secondary hypopituitarism usually persists. The disease can be resistant and recur despite appropriate treatment, especially in patients with risk factors. Therefore, long-term follow-up of patients is essential. Recurrent primary abscesses may require repeat EETS.

## Figures and Tables

**Fig. (2):**
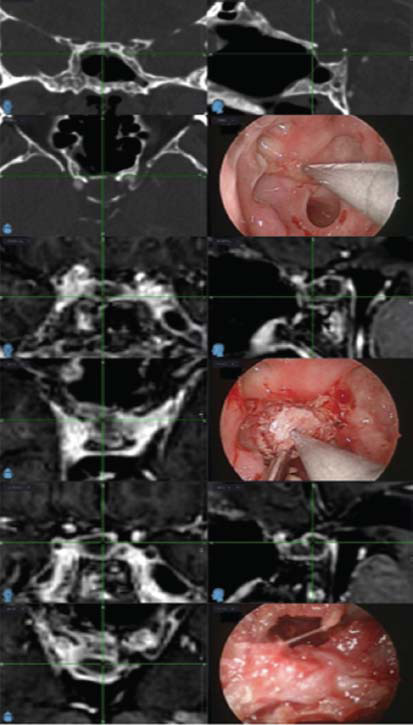
Screenshots are taken during the second ETSS procedure with guided MRI after abscess recurrence.

**Table 1:** Laboratory values showing anterior pituitary functions before and during follow-up.

Hormones	Initially	Postoperative 3. Month	Postoperative 1. Year
ACTH, NV 0-46 pg/ml	5.03	5.32	39.5
Cortisol, NV 6.24-18 μg/dl	0.95	0.49	2.7
TSH, NV 0.27-4.2 μIU/ml	4.43	0.01	0.01
FT_3_, NV 2.3-4.5 pg/ml	1.87	2.38	3.59
FT_4_, NV 0.93-1.7 ng/dl	0.29	1.67	1.68
GH, NV 0.126-9.88 ng/ml	0.18	0.26	0.37
IGF-1, NV* 37-219 μg/l	58.17	73.61	66.4
FSH, NV** 25.8-134.8 mIU/ml	1.42	1.23	3.3
LH, NV** 7.7-58.5 mIU/ml	<0.3	0.22	1.55
Estradiol, NV** <54.7 ng/l	<5	<5	<5
Prolactin, NV 6-29.9 ng/ml	164	10.7	69.1

**Abbreviations:** ACTH, adrenocorticotropic hormone; FSH, follicle stimulating hormone; FT3, free triiodothyronine; FT4, free thyroxine; GH, growth hormone; IGF-1, insulin-like growth factor 1; LH, luteinizing hormone; NV, normal values; TSH, thyrotropin. **Notes:** * The normal range for IGF-1 is given for age. ** Postmenopausal female normal ranges are given for FSH and LH.

## Data Availability

This article and its tables include all data obtained or analyzed as part of this study. The data archive can be made available on request. Further requests can be directed to the corresponding author.

## LIST OF ABBREVIATIONS

ACTH: Adrenocorticotropic Hormone
DI: Diabetes Insipidus
EETS: Endoscopic Endonasal Transsphenoidal Surgery
FSH: Follicle Stimulating Hormone
FT_3_: Free Triiodothyronine
FT_4_: Free Thyroxine
GH: Growth Hormone
IGF-1: Insulin-like Growth Factor 1
LH: Luteinizing Hormone
NV: Normal Values
TSH: Thyrotropin
MRI: Magnetic Resonance Imaging
W: Weighted

## STANDARDS OF REPORTING

CARE guidelines were followed.
